# Uncovering Symbionts Across the Psyllid Tree of Life and the Discovery of a New Liberibacter Species, “*Candidatus*” Liberibacter capsica

**DOI:** 10.3389/fmicb.2021.739763

**Published:** 2021-09-29

**Authors:** Younghwan Kwak, Penglin Sun, Venkata RamaSravani Meduri, Diana M. Percy, Kerry E. Mauck, Allison K. Hansen

**Affiliations:** ^1^Department of Entomology, University of California, Riverside, Riverside, CA, United States; ^2^Department of Botany, University of British Columbia, Vancouver, BC, Canada

**Keywords:** microbiome, *Candidatus* Liberibacter capsica, *Candidatus* Liberibacter psyllaurous, *Candidatus* Liberibacter asiaticus, endosymbiont, psyllid, endosymbiont evolution, long-term endosymbiont

## Abstract

Sap-feeding insects in the order Hemiptera associate with obligate endosymbionts that are required for survival and facultative endosymbionts that can potentially modify resistance to stress, enemies, development, and reproduction. In the superfamily Psylloidea, the jumping plant lice (psyllids), less is known about the diversity and prevalence of their endosymbionts compared to other sap-feeding pests such as aphids (Aphididae). To address this knowledge gap, using 16S rRNA sequencing we identify symbionts across divergent psyllid host lineages from around the world. Taking advantage of a new comprehensive phylogenomic analyses of Psylloidea, we included psyllid samples from 44 species of 35 genera of five families, collected from 11 international locations for this study. Across psyllid lineages, a total of 91 OTUs were recovered, predominantly of the *Enterobacteriaceae* (68%). The diversity of endosymbionts harbored by each psyllid species was low with an average of approximately 3 OTUs. Two clades of endosymbionts (clade 1 and 2), belonging to *Enterobacteriaceae*, were identified that appear to be long term endosymbionts of the psyllid families Triozidae and Psyllidae, respectively. We also conducted high throughput metagenomic sequencing on three *Ca*. Liberibacter infected psyllid species (*Russelliana capsici*, *Trichochermes walkeri*, and *Macrohomotoma gladiata*), initially identified from 16S rRNA sequencing, to obtain more genomic information on these putative Liberibacter plant pathogens. The phylogenomic analyses from these data identified a new *Ca*. Liberibacter species, *Candidatus* Liberibacter capsica, that is a potential pathogen of solanaceous crops. This new species shares a distant ancestor with *Ca*. L. americanus, which occurs in the same range as *R*. *capsici* in South America. We also detected the first association between a psyllid specializing on woody hosts and the Liberibacter species *Ca*. L. psyllaurous, which is a globally distributed pathogen of herbaceous crop hosts in the Solanaceae. Finally, we detected a potential association between a psyllid pest of figs (*M*. *gladiata*) and a *Ca*. Liberibacter related to *Ca*. L. asiaticus, which causes severe disease in citrus. Our findings reveal a wider diversity of associations between facultative symbionts and psyllids than previously reported and suggest numerous avenues for future work to clarify novel associations of ecological, evolutionary, and pathogenic interest.

## Introduction

The widespread application of genomic approaches has brought new insights to our understanding of the evolution of microbial symbioses with animals, especially for bacterial symbionts that are not culturable and have small genomes (Moran and Bennett, 2014). Many of these symbionts live inside the bodies or cells of herbivorous insects as symbionts and are involved in synthesizing nutrients, digesting and detoxifying plant materials and pesticides, and/or conferring defense toward natural enemies (Buchner, 1965; Moran, 2007; Hansen and Moran, 2014). These diverse insect-microbe associations, including some plant pathogens, enable insect herbivores to exploit different ecological niches and plant parts (Douglas, 2009; Feldhaar, 2011; Casteel and Hansen, 2014; Hansen and Moran, 2014; Sudakaran et al., 2017). These heritable symbiotic associations range from obligate mutualists and long-term endosymbiont associates that are generally present in all insect individuals of a species, to beneficial, commensal, and antagonistic facultative endosymbionts that are present in only some insect individuals of a population or species. Among plant sap-feeding herbivore groups with endosymbiotic associations, the pea aphids (Aphididae) and their endosymbionts are one of the most comprehensively studied (Oliver et al., 2010). Endosymbiont associations, however, are widespread in other pest sap-feeding insect systems as well. The relationships between these insect-symbiont systems are not as well-known, regardless of their putative impacts on insect phenotype and insect-plant interactions (Oliver et al., 2010; Feldhaar, 2011; Hansen and Moran, 2014; Sudakaran et al., 2017).

One group of sap-feeding herbivores, the psyllids in the superfamily Psylloidea, suborder Sternorrhyncha (Burckhardt et al., 2021) are known to harbor a diversity of heritable endosymbionts, which include obligate and facultative associations (Thao et al., 2000a; Hall et al., 2016; Morrow et al., 2017). Psyllids are also considered to be alternative hosts rather than passive carriers of the plant pathogen “*Candidatus* (*Ca*.) Liberibacter” (Nadarasah and Stavrinides, 2011); in turn *Ca*. Liberibacter is also regarded as a psyllid endosymbiont (Hansen et al., 2008; Casteel and Hansen, 2014; Hansen and Moran, 2014). Interestingly, *Ca*. Liberibacter only specializes in Psylloidea hosts and is not generally present in other insect superfamilies (Raddadi et al., 2011; Wang et al., 2017; Mauck et al., 2019). In consequence, new *Ca*. Liberibacter species such as “*Ca*. Liberibacter psyllaurous, *Ca*. L. europaeus, *Ca*. L. brunswickensis,” and “*Ca*. L. ctenaryainae” have been discovered in the past by screening psyllid species for microbes (Hansen et al., 2008; Raddadi et al., 2011; Morris et al., 2017; Thompson et al., 2017). This screening approach in psyllids not only adds to our understanding of endosymbiont associations that may impact psyllid fitness, but also identifies new *Ca*. Liberibacter species that may threaten our agriculture now, without our knowledge, or in the near future.

One of the first studies to reveal that psyllid species harbor a diversity of bacterial endosymbionts, in addition to its obligate nutritional endosymbiont “*Candidatus* Carsonella ruddii,” hereafter referred to as *Carsonella* (Baumann, 2005), was published by Thao et al. (2000a). Thao et al. (2000a) discovered that a large diversity of psyllid species harbor bacterial endosymbionts that belong to a wide diversity of taxonomic associations. Recently, Hall et al. (2016) and Morrow et al. (2017) investigated the diversity and composition of facultative endosymbionts by using conventional PCR and high-throughput 16S rRNA sequencing in a diversity of psyllid taxa from divergent families of Psylloidea, however, these studies primarily focused on native psyllid species from Australia. Thao et al. (2000a) and Morrow et al. (2017) found that most psyllid species harbor only one dominant facultative endosymbiont representative, in addition to *Carsonella*, suggesting that psyllids have a minimal core bacterial community. Moreover, in contrast to *Carsonella*, several studies have suggested that many of these psyllid endosymbionts were recently horizontally transmitted in psyllid taxa (Thao et al., 2000a; Spaulding and von Dohlen, 2001; Hall et al., 2016; Morrow et al., 2017). Currently, more studies are needed that examine a broader diversity of psyllid taxa from more geographic regions around the world to more fully understand the diversity, prevalence and codivergence patterns of psyllid endosymbionts among divergent psyllid lineages.

In addition to horizontally transmitted facultative endosymbionts, genomic studies on psyllid endosymbionts have revealed that some endosymbionts can have long-term evolutionary relationships with their psyllid host lineages (Sloan and Moran, 2012). In these cases, the long-term endosymbiont appears to be transitioning into an obligate endosymbiont role and is generally present in all individuals of that psyllid species (hereafter referred to as long-term endosymbiont). For example, these symbionts in transition display genomic signatures of long-term host associations, such as an increase in A + T richness of genomic sequences and genome reduction, while retaining genes that have a potential obligate role for their insect host (Sloan and Moran, 2012). Based on genome sequencing data, long-term endosymbionts in two psyllid species were identified with biosynthetic pathways for essential amino acid biosynthesis that complement pathways that are absent in *Carsonella* (Sloan and Moran, 2012). In another psyllid species, Nakabachi et al. (2013) identified *Ca*. Profftella armatura (hereafter referred as *Profftella*) in *Diaphorina citri* and revealed multiple roles of *Profftella* in providing defensive toxins, vitamins and carotenoids for the psyllid host. *Profftella* is also harbored in psyllid species that are closely related to *D*. *citri* (Nakabachi et al., 2020a,b). These results collectively suggest that some psyllid endosymbionts have a longer evolutionary association with certain psyllid lineages compared to more recent facultative endosymbiont associations.

To address multiple knowledge gaps regarding evolutionary associations among psyllids and their symbionts, we performed high-throughput 16S rRNA sequencing across 44 divergent psyllid species from 35 genera that reside in five of the seven families of Psylloidea, as classified by Burckhardt et al. (2021). These psyllids were collected from 11 different geographic regions around the world. This wide sampling approach, both taxonomically and geographically, allows us for the first time to (1) identify endosymbiont associations, including novel *Ca*. Liberibacter species, for 44 divergent psyllid taxa, and (2) detect hallmark signatures of long-term endosymbiont evolution to identify putative long-term endosymbiont taxa in psyllids. In addition, we (3) determine the phylogenetic relationship of all new *Ca.* Liberibacter taxa identified in psyllid microbiomes using gene content from metagenomic sequencing.

## Materials and Methods

### Psyllid Collection and DNA Extractions

For 16S rRNA amplicon sequencing (detailed below), a total of 45 psyllid samples were analyzed, and included 44 different psyllid species. Two of the psyllid samples were the same psyllid species, *Trioza urticae*, however, the samples were collected from two different locations and time points (Supplementary Table 1). The 44 psyllid species represent 35 genera from five families within Psylloidea. Psyllid species were collected from 11 international geographic locations for this study and are detailed in Supplementary Table 1. Each psyllid sample consisted of an average of two psyllid adults pooled for DNA extractions, because most psyllid species are extremely small and not enough DNA can be obtained from just one individual per sample given our extraction protocol, which preserves voucher specimens for species identification of each sample (see below). Only the *Trichochermes walkeri* (P1–28) sample consisted of pooled nymphs (Supplementary Table 1). Psyllid DNAs were extracted following a nondestructive DNA extraction protocol (Percy et al., 2018) in order to correctly identify psyllid species with high confidence using both a molecular and morphological diagnostic approach as described in Percy et al. (2018). Briefly, collections were made by sweep net and aspirating directly from the plant, specimens were placed into 95–100% ethanol in the field and stored at −20°C thereafter. Field collected specimens were sorted and identified by DP and are maintained in DP’s personal collection; individuals selected for DNA extraction were then placed (whole or bisected) in the tissue lysate buffer with proteinase K [as per the Qiagen (Germantown, Maryland) protocol] and incubated for 12–15 h at 56°C. After incubation the specimen was retained and preserved in 70–95% ethanol as a DNA voucher. Genomic DNAs was obtained using a Qiagen DNeasy Blood and Tissue Kit and eluted in 200 μl of the elution buffer.

### High Throughput Sequencing and Data Analysis of 16S rRNA Amplicons

For library preparation of 16S rRNA libraries, custom 16S rRNA primers were synthesized by Integrated DNA Technologies, Inc. (San Diego, CA, United States) using a dual barcode design with 25 primer pairs based on the primer constructs used by the Earth Microbiome Project (EMP^1^) similar to Walters et al. (2016) and Caporaso et al. (2012). Our aim was to amplify the 16S rRNA gene region of endosymbionts from psyllids including putative plant pathogens such as *Ca*. Liberibacter species. In turn, these barcode primers contained the primer sequences (341F and 805R) that target the 16S rRNA region. The primer sequences (341F and 805R) were designed previously by Morrow et al. (2017), because these primers successfully amplified psyllid endosymbionts in addition to plant pathogens such as *Ca*. Liberibacter species; the forward primer, 16S-341F = 5′-CCTACGGGNGGCWGCAG-3′, and the reverse primer, 16S-805R = 5′-GACTACHVGGGTATCTAATCC-3′. These latter primers span the V3–V4 region of the 16S rRNA gene resulting in a 464 bp amplicon. This primer set was selected, based on results from Morrow et al. (2017), because it did not preferentially amplify the most abundant obligate endosymbiont of psyllids, *Carsonella*. *Carsonella* is present in all psyllids at very high titers (Thao et al., 2000b). Moreover, these primers did not preferentially amplify plant plastid 16S rRNA genes from psyllid DNA (Morrow et al., 2017), which can abundantly amplify from psyllid DNA when targeting other variable regions of the 16S rRNA gene (Nachappa et al., 2011).

Amplicon PCR using the above primer set consisted of 4 μl of template DNA, 0.9 μl of each 10 μM primer, and 20 μl of Phusion master mix (ThermoFisher Scientific) per each reaction. The 16S rRNA amplicons were then purified using PureLink^TM^ PCR Purification Kit (Invitrogen). Using the cleaned PCR product as template, a second PCR was performed with HPLC-cleaned primers (5′-TATGGTAATTGTCCTACGGGNGGCWGCAG-3′ and 5′-AGTCAGCCAGCCGACTACHVGGGTATCTAATCC-3′) to complete the Illumina sequencing construct as described in McFrederick and Rehan (2016). Second PCR reactions consisted of 1 μl of template DNA, 0.5 μl of each forward and reverse primer, and 23 μl of Phusion master mix (ThermoFisher Scientific). For all PCR conditions, we followed PCR protocols as described in McFrederick and Rehan (2016). A total of 18 μl of PCR product from each reaction was normalized using SequalPrep^TM^ Normalization Plate Kit (Invitrogen). We pooled 4 μl of each of the normalized samples, and performed additional bead clean up. The quality of the libraries was then assessed using a 2100 Bioanalyzer.

All 16S rRNA amplicons from psyllid samples were multiplexed in a single lane for dual index sequencing on the Illumina MiSeq PE300 at the DNA Technologies and Expression Analysis Core Laboratory at the University of California, Davis. The mothur v.1.41.3 pipeline was used to process raw read data following the protocol described in the MiSeq Standard Operating Procedure to process high-quality contigs from overlapping paired-end reads (Schloss et al., 2009; Kozich et al., 2013). To trim off adaptors and control against sequencing errors, low-quality reads were trimmed and removed using the following parameters: the maximum ambiguous base = 0, the maximum sequence length = 440 bp. Chimera vsearch was then used to remove chimeric sequences. To assign taxonomy, the SILVA database (release 132) was used. Operational taxonomic units (OTUs) were clustered at the level of ≥ 97% nucleotide sequence similarity (Stackebrandt and Goebel, 1994).

Morrow et al. (2017) suspected a high amount of OTU splitting when using the primers 341F and 805R (see above) because several OTUs were highly similar in SILVA taxonomy affiliation within a single psyllid sample. These related OTUs potentially can be real and represent different co-occurring strains within a sample, however, they can also represent amplification and/or sequencing error (during library preparation and/or Illumina sequencing), or different 16S rRNA copies from a single bacterium. To help control further against amplification and sequencing errors, in addition to the read quality control filtering steps mentioned above, OTUs with low read abundance per sample were removed from the dataset during the OTU picking step if the total counts of contigs for a particular OTU were equal to and/or less than five. Moreover, to control against environmental or aerosolized contaminates during library preparation five control samples that consisted of water instead of the DNA template were also included during the construction of the 16S rRNA libraries to detect if library contamination occurred. After taxonomy assignments using SILVA (above) we used BLASTn searches against the NCBI non redundant nucleotide database to further annotate 16S rRNA contigs. Furthermore, the aim of this study was to identify common and abundant endosymbionts and plant pathogens within psyllid bodies, therefore, OTUs of mitochondria, chloroplasts, and *Carsonella* were removed prior to analysis.

### A + T Richness Analyses of Operational Taxonomic Unit 16S rRNA Gene Sequences

The 16S rRNA gene sequences of known free-living bacterial relatives or endosymbionts belonging to three host association categories such as obligate, long-term symbionts that co-occur with obligate endosymbionts, and facultative endosymbionts were obtained from NCBI GenBank to compare AT/GC content with the same 16S rRNA region of sequences in this study (Supplementary Table 2). All NCBI sequences were aligned with the 16S sequences here to obtain the same 16S rRNA gene region using NCBI BLASTn. The percent GC nucleotide composition was calculated for each of these aligned 16S rRNA sequence regions using BBMap (Bushnell, 2014) and Genomics % G–C Content Calculator (Science Buddies 2021). The box and whisker plot were generated based on GC content data using the package “ggplot” (Wickham, 2016) in R v.4.0.2 integrated in Rstudio v.1.3.959 (R Core Team, 2021).

### Metagenomic Sequencing and Data Analysis of *Ca*. Liberibacter Infected Samples

Psyllid samples where *Ca*. Liberibacter was identified, using 16S rRNA sequencing (above), were further examined using a metagenomic approach. The goal of metagenomic sequencing was to generate more sequence data for *Ca*. Liberibacter loci, to further determine the evolutionary relationships of newly identified *Ca*. Liberibacter taxa. Psyllid samples that were positive for *Ca*. Liberibacter, based on 16S rRNA sequencing, include *Macrohomotoma gladiata* (P1–9), *Russelliana capsici* (P1–16), and *Trichochermes walkeri* (P1–28). A preserved herbarium specimen of *Solanum umbelliferum* (HS9) collected in 2011 that was infected with the *Ca*. L. psyllaurous haplotype G, based on our previous study Mauck et al. (2019), was also sequenced. Prior to library preparation, each sample was subjected to whole genome amplification using the Repli-G whole genome amplification kit (Qiagen) according to the manufacturer’s instructions. Illumina library preparation and sequencing were conducted by Vincent J. Coates Genomics Sequencing Laboratory at the University of California, Berkeley. To increase the number of reads per sample, each *Ca*. Liberibacter-infected psyllid/plant library was sequenced twice as paired-end, 150 bp reads on two lanes of Illumina NovaSeq SP. The quality of raw reads for each sample run was verified with FASTQC v.0.11.3 (Andrews, 2010), and low-quality reads and adapter sequences were removed using Trimmomatic v.0.36 (Bolger et al., 2014). For each independent sample run, the trimmed sequences were assembled using MEGAHIT v.1.2.8 (Li et al., 2015), and the resulting contigs were categorized into either unassigned taxonomy or *Ca*. Liberibacter based on best BLASTn hits against nucleotide databases. To assess the overall quality of *Ca*. Liberibacter metagenomes, read coverage statistics of putative *Ca*. Liberibacter associated contigs were calculated using BBMap (Bushnell, 2014). To improve the binning of *Ca*. Liberibacter specific contigs, contigs from assembly outputs of each independent sample run were analyzed following the Anvi’o metagenomic workflow (Eren et al., 2015). Putative *Ca*. Liberibacter contigs were selected from both Anvi’o and best BLAST hit results from NCBI BLASTn with the following constraints, *E*-value = 1e-4 and sequence similarity >70%. Resulting putative *Ca*. Liberibacter contigs from independent sample runs were merged for each sample using MEGAHIT v.1.2.8. (Li et al., 2015). The genome assemblies were annotated using Prokka v1.14.5 (Seemann, 2014), and BLASTp searches of output files were conducted against non-redundant NCBI databases.

### Phylogenetic Analyses

To determine the evolutionary relationships of putative novel *Ca*. Liberibacter species, orthologous proteins were obtained from metagenomes generated in this study and those available on NCBI Genbank (below) using Orthofinder (Emms and Kelly, 2015). For phylogenetic analyses, we included a total of four draft *Ca.* Liberibacter genomes from this study and seven complete *Ca*. Liberibacter reference genomes from NCBI of *Ca*. L. africanus (NZ_CP004021.1), *Ca*. L. americanus (NC_022793.1), *Ca*. L. asiaticus (NC_012985.3), *Ca*. L. europaeus (GCA_003045065.1), *Ca*. L. psyllaurous (NC_014774.1), and *L*. *crescens* (NC_019907.1) in phylogenetic analyses. Due to low read coverage of *Ca*. Liberibacter in one of the *Ca*. Liberibacter positive psyllid samples (*M*. *gladiata*, P1–9) a low number of shared orthologs were identified. In consequence, phylogenetic analyses were conducted only with samples obtained from the psyllids *R*. *capsici* (P1–16) and *T*. *walkeri* (P1–28), in addition to the herbarium sample, *S*. *umbelliferum* (HS9), which shared 102 orthologs with the six fully sequenced *Ca*. Liberibacter reference genomes (above; Supplementary Table 3).

For the ortholog phylogenetic analysis, 102 orthologous sequences from three draft genomes along with the reference sequences (see above) were aligned using MAFFT v7.407 (Katoh and Standley, 2013). Trimming was performed using trimAL v1.2 (Capella-Gutiérrez et al., 2009) with two parameters, gap threshold “gt” and minimum coverage: “cons” in the trimmed alignment. In this case the gt and cons were considered as 0.9 and 20, respectively. All the alignments were visualized using the graphical user interface, and the PHYLIP file was generated from Mesquite v3.51 (Maddison and Maddison, 2018) and further used for phylogenetic tree construction. RAxML version 8.2.12 (Stamatakis, 2014) was used to obtain phylogenetic inferences. The MPI version was executed to run the program parallelly over many connected machines on the cluster. The “PROTGAMMAAUTO” model along with option (-f a) was used to perform rapid bootstrap analysis and to find the best scoring Maximum Likelihood (ML) tree. 100 searches from the parsimony start tree were performed. The Bipartitions tree generated from RAxML was visualized using FigTree v1.4.4 (Rambaut, 2018). The generated tree was then exported to Adobe Illustrator v 25.1 (Adobe Systems Incorporated, United States) for further editing.

For the 16S rRNA and endosymbiont phylogenetic analyses, similar to above, *Ca*. Liberibacter and endosymbiont sequences from 16S rRNA gene amplicon sequencing and NCBI GenBank were aligned using MAFFT v7.407 (Katoh and Standley, 2013) and trimmed manually. Mesquite v3.51 (Maddison and Maddison, 2018) was then used to generate the PHYLIP file. To generate the ML tree, the file was submitted to the CIPRES web server to run the RAxML-HPC BlackBox with “GTRGAMMA” model. For the *Ca*. Liberibacter, *Enterobacteriaceae*, or *Wolbachia* phylogeny the outgroup was set as *L*. *crescens*, *Escherichia coli* strain U 5/41, and *Wolbachia* endosymbiont of *Cimex lectularius*, respectively, and other settings were kept as default. Visualization and further editing of the bipartitions tree was performed using FigTree v1.4.4 (Rambaut, 2018) and Adobe Illustrator v 25.1 (Adobe Systems Incorporated, United States). The Psylloidea cladogram of 44 psyllid species, that was modified from Burckhardt et al. (2021), was redrawn using the package “ape” (Paradis et al., 2004) in R v.4.0.2 integrated in Rstudio v.1.3.959 (R Core Team, 2021).

### DNA–DNA Hybridization and Average Nucleotide Identity Analyses

If phylogenetic analyses and BLAST identify a potentially new *Ca*. Liberibacter species DNA--DNA hybridization (DDH) and the average nucleotide identity (ANI) values will be computed pairwise with the new *Ca*. Liberibacter species and the reference *Ca*. Liberibacter genomes used above for phylogenetic analyses. DDH values were calculated using the Genome-to-Genome Distance Calculator 3.0 (GGDC^2^)(Meier-Kolthoff et al., 2013). Briefly, according to Meier-Kolthoff et al. (2013) genome distances were calculated with BLAST+ local alignments and by calculating genomic distance as identities/HSP length (Formula 2). Distance values were then converted to estimated DDH values and their associated confidence intervals using a generalized linear model. The ANI of common genes and the percentage of conserved DNA among the *Ca*. Liberibacter genomes were calculated using the ANI calculator^3^ (Rodriguez and Konstantinidis, 2016).

### Further Screening for *Ca*. Liberibacter Taxa in Additional Psyllid Specimens

We obtained additional psyllid specimens from species that were identified as *Ca*. Liberibacter positive from our 16S rRNA amplicon sequencing to determine how prevalent *Ca*. Liberibacter infection is in these species. We were able to obtain additional psyllid specimens from two of the three *Ca*. Liberibacter positive species, which includes *Trichochermes walkeri* (*N* = 9 samples) and *Macrohomotoma gladiata* (*N* = 2 samples) (Supplementary Table 4). We also obtained three additional psyllid species to screen that feed on the same host plant genus (*Rhamnus* sp.) as one of our *Ca.* Liberibacter infected species here, *T*. *walkeri*, to determine if these psyllid species were infected with *Ca.* Liberibacter as well; these psyllid species include *Trioza rhamni* (*N* = 4 samples), *Cacopsylla alaterni* (*N* = 6 samples), and *Cacopsylla rhamnicola* (*N* = 7 samples) (Supplementary Table 4). Depending on specimen availability, for each psyllid sample a single adult individual and/or 2–3 nymphs were pooled for each DNA extraction (Supplementary Table 4).

DNA was extracted from whole psyllids using the Quick-DNA^TM^ Microprep Kit (Zymo Research). DNA quality and yield was assessed by gel electrophoresis and the SpectraMax QuickDrop Micro-Volume Spectrophotometer (Molecular Devices). Based on *Ca*. Liberibacter phylogenetic analyses here of 16S rRNA amplicon samples (see above), *T*. *walkeri* and *M*. *gladiata* were infected with *Ca*. Liberibacter taxa that were closely related to *Ca*. L. asiaticus and *Ca*. L. psyllaurous, respectively, in consequence, modified universal *Ca*. Liberibacter primers, LG774F/LG1436R (Morris et al., 2017), were used for screening additional psyllid specimens. This primer pair is expected to amplify a 684 bp fragment from the 16S rRNA gene region of *Ca*. L. asiaticus, *Ca*. L. psyllaurous, and *Ca*. L. europaeus. PCR was performed in 25 μl reactions consisting of 2 μl of DNA template, 2.5 μl of 10X Thermopol buffer (NEB), 0.5 μl of dNTP mix (2 mM each), 0.5 μl of each 10 μM primer, and 0.125 μl of Taq DNA polymerase (NEB). The Bio-Rad C1000 Touch^TM^ Thermal Cycler was used with the following protocol: 95°C for 30 s, followed by 30 cycles of 95°C for 30 s, 58°C for 1 min, and 72°C for 1 min, then 72°C for 5 mins. Only *T*. *walkeri* samples (*N* = 9 samples) were positive for *Ca*. Liberibacter based on PCR screening with universal *Ca*. Liberibacter primers (above). In turn, amplicons from *T*. *walkeri* were purified with QIAquick Gel Extraction Kit (Qiagen) and cloned into the pGEM T-easy vector (Promega) following the manufacturer’s instruction. Sanger sequencing was conducted by the Genomics Core facility at the University of California, Riverside.

## Results

### 16S rRNA Amplicon Sequencing of Psyllid Microbiomes

Amplicon sequencing of the bacterial 16S rRNA gene region from 45 psyllid samples, which includes 44 psyllid species (Supplementary Table 1), generated a total of 7,516,747 contigs. Following quality control and criteria filtration, 198,560 high-quality contigs (305,128 reads) were retained. The total number of contig counts per psyllid sample ranged from 17 to 4,531 with an average of 798. These contigs were clustered into 91 OTUs, and each psyllid sample possessed an average of 2–3 OTUs, (min = 1, max = 7, and SD = 1; Supplementary Table 5). Non-target OTUs, such as mitochondria, chloroplasts, and *Carsonella*, only represented 7% of the total reads for high-quality contigs. Seven OTUs were present in two out of the five control samples. Specifically, three OTUs were unique to one control sample (P2–96) and four OTUs were unique to the second control sample (P3–96) (Supplementary Table 6). None of the OTUs in the control samples were present in any of the psyllid samples. The contig counts for control samples were relatively low with an average of 21 contigs per OTU (min = 7, max = 80, and SD = 27) (Supplementary Table 6). In turn, sequences present in control samples may have been airborne contaminants that were present at low abundance during the library preparation process.

### Low Diversity of Putative Psyllid Endosymbiont Operational Taxonomic Units Within 44 Divergent Psyllid Species From 45 Psyllid Samples

**FIGURE 1:**
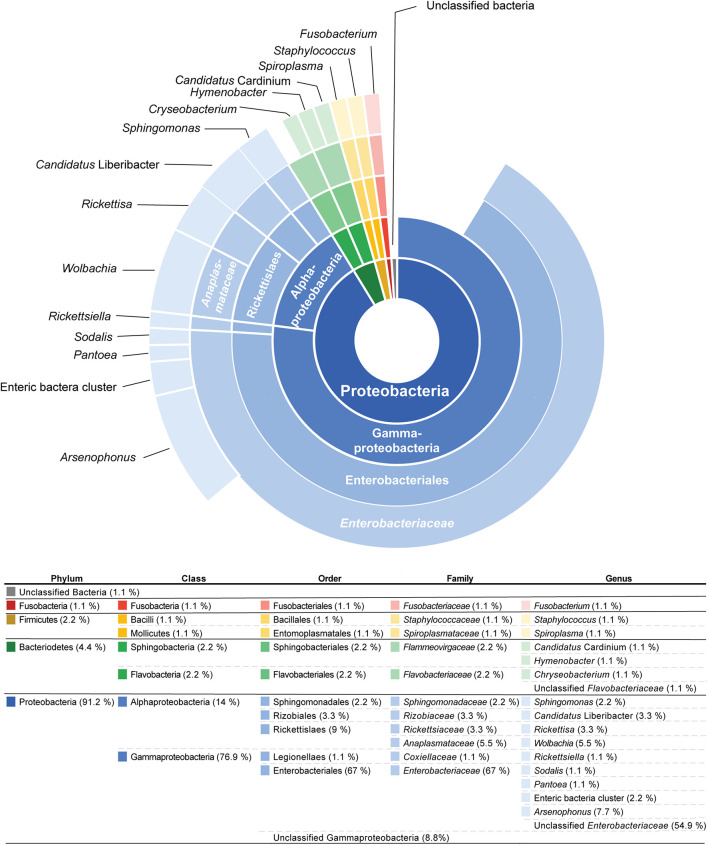
The sunburst diagram above represents the percentage of 91 operational taxonomic units (OTUs) from 44 psyllid species (from 45 psyllid samples) that belong to a particular taxonomic group. The ring positions of the diagram represents taxonomic hierarchy: (inner to outer) Phylum/Class/Order/Family/Genus.

From 16S rRNA amplicon sequencing, OTUs that belong to four bacterial phyla (Proteobacteria, Bacteroidetes, Firmicutes, and Fusobacteria) were identified from 45 psyllid microbiomes (Figure 1). The majority of OTUs, among all psyllid samples examined, reside within Proteobacteria (83 OTUs out of 92 OTUs) (Supplementary Table 5), and all psyllid samples possess Proteobacteria OTUs (Supplementary Table 5). Within Proteobacteria, OTUs reside from two classes, Alphaproteobacteria (13 OTUs) and Gammaproteobacteria (70 OTUs), and were found in 24 and 36 psyllid samples, respectively (Supplementary Table 5).

The majority of OTUs (77%) from psyllid samples belong to the class Gammaproteobacteria, and primarily reside within the bacterial Class *Enterobacteriaceae* (68% of OTUs) (Figure 1). These *Enterobacteriaceae* OTUs were unique to their psyllid species, except for OTUs 8, 33, 72, 76, and 162, which occur in more than one psyllid sample (Supplementary Table 5 and Supplementary Figure 1). Approximately 55% of *Enterobacteriaceae* OTUs were not identified to the genus level, however, ∼8, 1, 1, and 2% were identified as *Arsenophous*, *Sodalis*, *Pantoea*, and Enteric bacteria, respectively (Figure 1 and Supplementary Table 5).

Within the class Alphaproteobacteria, five *Wolbachia* (*Anaplasmataceae*) OTUs were present in 19 out of 45 psyllid samples (∼42% of all psyllid samples). *Wolbachia* OTU1 was present in 19 psyllid samples/species (Supplementary Figure 1 and Supplementary Table 5), and the other four *Wolbachia* OTUs co-occur with *Wolbachia* OTU1 in three psyllid samples, *Cornegenapsylla allophyli*, *Cacopsylla pruni*, and *Trioza vitiensis* (Supplementary Table 5). Other alphaproteobacterial OTUs include the genus *Sphingomonas* and *Rickettsia* (Figure 1). The genus *Spingomonas* was detected in four psyllid samples (∼9% of all psyllid samples) with two different OTUs; OTU1799 was detected in *Pachypsylla celtidismamma*, while OTU72 was present in *Livia junci*, *Triozoida limbata*, and *Arytaina devia* ssp. *insularis* (Supplementary Table 5 and Supplementary Figure 1). The psyllid sample, *C*. *pruni*, was infected with two different *Rickettsia* OTUs, OTU127 and OTU367, and *Heterotrioza chenopodii* was detected with *Rickettsia* OTU183 (Supplementary Table 5).

BLAST results from psyllid microbiome 16S rRNA sequences reveal that the vast majority (80%) of sequences had best BLAST hits to insect endosymbionts on NCBI GenBank and displayed an average of >∼96% nucleotide sequence similarity to known insect-associated endosymbionts (Supplementary Table 5). The remaining sequences were primarily found to be plant (2%) or environmental associated microbiomes (10%) (Supplementary Table 5). Sequences that are related to known plant pathogens were ∼5%, including the three *Ca*. Liberibacter OTUs and a Xylella related OTU (OTU155) (Supplementary Table 5). Only two OTUs (OTUs 618 and 1,355) (2%) in two samples had best BLAST hits to human associated microbes, and therefore these latter OTUs may be contamination from psyllid collection or extraction (Supplementary Table 5).

### Identification of Putative Long-Term Endosymbionts in Psyllid Species

**FIGURE 2:**
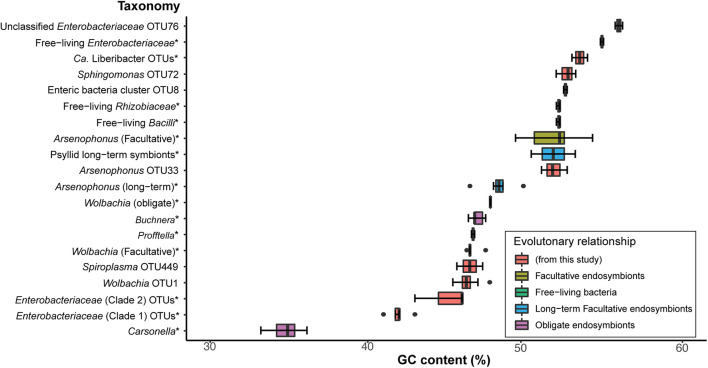
Comparison of GC contents from a 429-nt 16S rRNA gene region obtained from endosymbiont taxa of known insect host associations (obligate, long-term, and facultative), free-living bacterial relatives, and a selection of OTUs from this study. * indicates that detailed OTU and NCBI sequence and species information is available in Supplementary Table 2. Percent GC content for all 16S rRNA sequence data are also presented in Supplementary Table 2. Box and whisker plots represent average percent GC content and the standard deviation.

The obligate endosymbiont of aphids (*Buchnera*), in addition to other obligate host-restricted endosymbiont species in insects, possess 16S rRNA gene sequences that are significantly higher in AT richness compared to their free-living relatives (Lambert and Moran, 1998; Nováková et al., 2009). Therefore, we analyzed GC content of the 16S rRNA gene region sequenced for OTUs in this study (Supplementary Table 2) and compared them to free-living relatives and known obligate, long-term, and facultative endosymbionts of insects. These free-living relatives and known insect endosymbionts were chosen because they belong to the same bacterial classes that the majority of OTUs from our study were classified as based on Silva, as average GC content can vary with taxonomy (Supplementary Table 2). Specifically, we analyzed: (1) the percentage of GC content from 16S rRNA sequences and (2) the phylogenetic patterns of 16S rRNA sequences that were in the average GC percent range of known primary and long-term insect endosymbionts of insects (Figure 2).

We found that *Carsonella*, the obligate endosymbiont of psyllids, displays the lowest average GC content (∼35% GC and SD = 0.9) compared to all sequences analyzed in this analysis for the same 402-nt 16S rRNA gene region (Supplementary Table 2 and Figure 2). In contrast, the obligate endosymbiont of aphids, *Buchnera*, which also belongs to Gammaproteobacteria, shows an average of ∼47% GC (SD = 0.3) content (Figure 2), which was very similar to *Profftella*, the Gammaproteobacterial long-term symbiont that is harbored within the psyllid genus Diaphorina (Figure 2 and Supplementary Table 2). Other Gammaproteobacterial long-term endosymbionts identified in the psyllids *Ctenarytaina eucalypti* and *Heteropsylla cubana* displayed an average of ∼52% GC content (Figure 2 and Supplementary Table 2). Obligate and long-term symbionts that belong to Alphaproteobacteria and Gammaproteobacteria such as *Wolbachia* and *Arsenophonus*, respectively, displayed an average of ∼48% (SD = 0 and 0.9, respectively) GC content for this 16S rRNA gene region (Figure 2 and Supplementary Table 2). Interestingly, facultative *Wolbachia* endosymbionts display very similar average GC content (∼ 47% GC, SD = 0.4) compared to the average of obligate *Wolbachia* endosymbionts. In contrast, facultative *Arsenophonus* symbionts displayed an average of ∼52% GC (SD = 1.6) content (Figure 2 and Supplementary Table 2). The free-living relatives of these latter endosymbionts and sequences from this study display an average of ∼53% GC (SD = 1.4), which is in the range of several facultative endosymbionts analyzed in this study (Figure 2 and Supplementary Table 2). Overall, we found that the average percentage of GC content of obligate and long-term endosymbionts ranged from ∼41 to 49% GC (Figure 2 and Supplementary Table 2).

**FIGURE 3:**
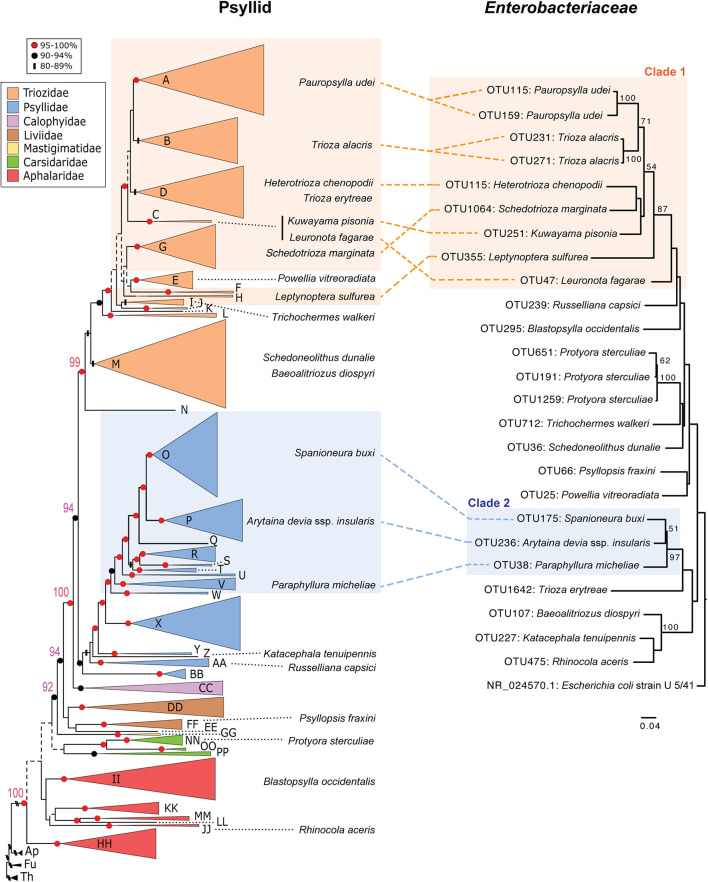
Comparison of phylogenies from both psyllid hosts and their endosymbiont *Enterobacteriaceae*. Maximum likelihood tree of Psylloidea (*left*) redrawn from Percy et al. (2018) with updated classification according to Burckhardt et al. (2021). For the psyllid phylogeny node support is indicated by shape and color of node symbols, and nodes with <50% bootstrap support are indicated by dotted lines. Capital letters in the psyllid phylogeny represent generic groups presented in Percy et al. (2018) and are detailed in Supplementary Table 1. Psyllid species names from samples in this study that harbor *Enterobacteriaceae* with 16S rRNA sequences that have a GC content in the range of ∼41–49% are indicated on both the psyllid and *Enterobacteriaceae* phylogenies. For the *Enterobacteriaceae* 16S rRNA phylogeny (429-nt) using RAxML with 100 bootstraps only branch support at 50% or above is shown. The scale bar indicates nucleotide changes per site. The tree was rooted with the outgroup *Escherichia coli* strain U 5/41 (NR_024570.1).

A second signature of long-term endosymbiont evolution is congruent phylogenetic patterns between endosymbiont insect taxa and their insect hosts. For this analysis we only constructed phylogenies of endosymbiont sequences if their 16S rRNA sequence’s percent GC content was between the average range of obligate and long-term endosymbionts (∼41–49%) (above). We found 34 sequences that fell within this range and they either belonged to *Enterobacteriaceae* or *Wolbachia*. In turn we constructed two separate phylogenies for these two distinct classes of bacteria (Figure 3 and Supplementary Figure 2). Compared to the psyllid host phylogeny, *Wolbachia* sequences appear to be recently diverged, largely sharing the same OTU (OTU1) and are horizontally transmitted across all psyllid families examined (Supplementary Figure 2). For the *Enterobacteriaceae* phylogeny four distinct bacterial lineages were supported with bootstrap values of 87% and higher. However, only two clades (hereafter named clade 1 and 2) appear to have congruent phylogenies with the psyllid host phylogeny at the family level for Triozidae and Psyllidae (Figure 3). More sequence information for both bacterial and psyllid phylogenies are required, however, to provide robust bootstrap support at a finer resolution.

### *Candidatus* Liberibacter Taxa Identified From 16S rRNA Amplicon Sequencing in Three Psyllid Species

Three *Ca*. Liberibacter OTUs (OTU526, OTU841, and OTU443) were identified in three different psyllid species, *Macrohomotoma gladiata* (P1–9), *Russelliana capsici* (P1–16), and *Trichochermes walkeri* (P1–28). In *M*. *gladiata*, OTU526 has the best BLAST hit to *Ca*. L. asiaticus with 98% nucleotide sequence similarity (Supplementary Table 5). The OTU841 in *R*. *capsici* has the best BLAST hit to *Ca*. L. americanus with 96.8% nucleotide sequence similarity (Supplementary Table 5). The OTU443 from the psyllid sample *T*. *walkeri*, displayed the best BLAST hit to “unclassified *Ca*. Liberibacter” with 98.3% nucleotide sequence similarity (Supplementary Table 5). To further determine the identity and evolutionary relationships of these latter *Ca*. Liberibacter taxa psyllid DNA samples that were positive for *Ca*. Liberibacter were subject to metagenomic sequencing for downstream phylogenetic analyses.

### Four *Ca*. Liberibacter Draft Genomes Were Obtained From Metagenomic Sequencing

To further analyze *Ca*. Liberibacter taxa identified from 16S rRNA amplicon sequencing, we performed metagenomic sequencing on the three *Ca*. Liberibacter positive psyllid samples (*M*. *gladiata*, *R*. *capsici*, and *T*. *walkeri*) and one herbarium specimen infected with *Ca*. L. psyllaurous haplotype G from a previous study (Mauck et al., 2019). An average of 66,126,618 high quality reads were generated for each independent psyllid/plant sample run (SD = 5,675,882; Supplementary Table 7). Metagenomic assemblies for the four samples yielded an average of ∼0.9 Mb (SD = 0.3 Mb) of *Ca*. Liberibacter specific sequences per sample with approximately 322–1,637 contigs per sample (Table 1). The *Ca*. Liberibacter assembly from *M*. *gladiata* had very low sequence coverage and a low N50 value (Table 1) and therefore was not included in downstream phylogenetic analyses. The remaining three *Ca*. Liberibacter assemblies had variable levels of read coverage per contig because DNA samples were of low concentration and obtained from Repli-G whole genome amplification (Supplementary Table 8), however, a total of 102 shared orthologous *Ca*. Liberibacter genes (Supplementary Table 3) were still obtained and used for subsequent phylogenetic analyses.

**TABLE 1:** Statistics of mapping and assembly of *Ca*. Liberibacter sequences from psyllids and plant DNA.

**Samples**	***M*. *gladiata***	***R*. *capsici***	***T*. *walkeri***	***S*. *umbelliferum***
Total sequence length (bp)	606681	1212287	922660	803932
The number of contigs	1,063	1,637	322	407
N50 (bp)	583	1,530	4,943	3,031
Average (bp)	570	740	2,865	1,975
Min (bp)	200	200	301	301
Max (bp)	6,149	23,655	17,720	15,041

### New *Ca*. Liberibacter Taxa Uncovered by Phylogenetic, DNA–DNA Hybridization, and Average Nucleotide Identity Analyses

**FIGURE 4:**
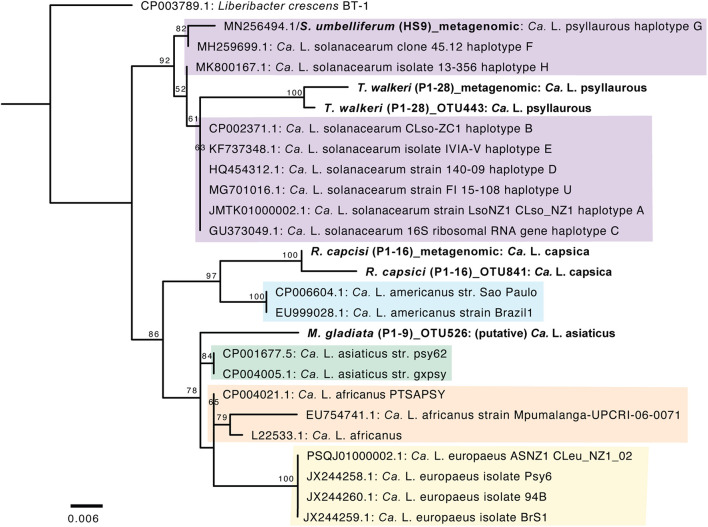
Phylogenetic relationships of *Ca*. Liberibacter species based on a 402-nt 16S rRNA region using RAxML with 100 bootstraps. The branch labels in bold are 16S rRNA sequences obtained here from 16S rRNA amplicon sequencing (indicated with OTU numbers) and metagenomic sequencing (indicated as metagenomic). The tree was rooted with the outgroup *Liberibacter crescens* BT-1. Only branch support at 50% or above is shown. Scale bar indicates nucleotide changes per site.

A phylogenetic analysis was first conducted with *Ca*. Liberibacter 16S rRNA gene sequences that were derived from both 16S rRNA amplicon sequencing and metagenomic sequencing. The *Ca*. Liberibacter species from *T*. *walkeri* (P1–28) formed a clade together within other *Ca*. L. psyllaurous taxa with 92% bootstrap support (Figure 4). The *Ca*. Liberibacter species from *R*. *capsici* (P1–16) forms a distinct lineage apart from other *Ca*. Liberibacter species (Figure 4). This latter *Ca*. Liberibacter taxa shares a more recent common ancestor with *Ca*. L. americanus compared to other *Ca*. Liberibacter species (Figure 4). Only the 16S rRNA sequence of *Ca*. Liberibacter from *M*. *gladiata* (P1–9) was obtained through 16S rRNA amplicon sequencing and this sequence appears to be more closely related *Ca*. L. asiaticus (Figure 4 and Supplementary Table 5). The sequence divergence between the 16S PCR-derived sequence and the metagenomic-derived 16S sequence for both P1–16 and P1–28 was present, however, bootstrap support was very high (100%) and nucleotide differences were very small (as the scale on the figure indicates 0.006 nucleotide substitutions per site). This sequence divergence between PCR and metagenomic 16S sequences may be attributed to sequencing error or nucleotide substitutions due to multiple 16S rRNA operons per Liberibacter genome and/or co-occurring strains in pooled psyllid samples.

**FIGURE 5:**
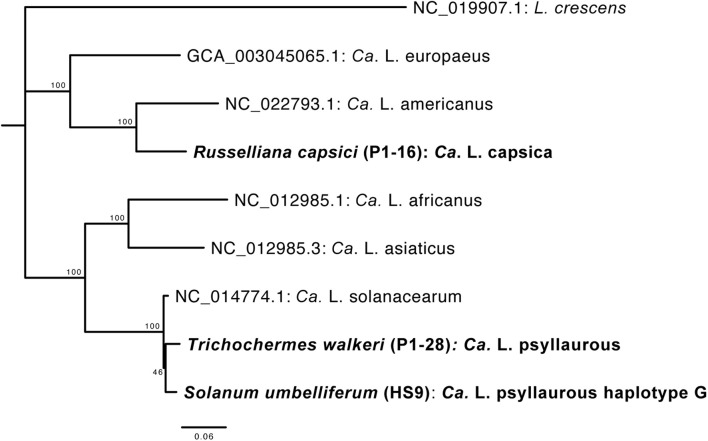
Phylogenetic relationships of *Ca*. Liberibacter species based on a 27,922 amino acid alignment of 102 orthologous genes using RAxML with 100 bootstraps. Orthologs from the *Ca*. Liberibacter taxa were obtained from three draft metagenomes sequenced here and are shown in bold with their psyllid host names indicated. The tree was rooted with the outgroup *Liberibacter crescens*. Scale bar indicates amino acid changes per site.

Since the latter 16S rRNA phylogenetic analysis was constrained in only using a 402 nt sequence region, which was amplified by 16S amplicon sequencing, a more robust phylogenetic analysis was subsequently conducted. The second phylogenetic analysis consists of a 27,922 amino acid alignment of 102 orthologous proteins. From this analysis the *Ca*. Liberibacter species from *R*. *capsici* (P1–16) forms a distinct clade apart from the other *Ca*. Liberibacter species based on the high amount of amino acid changes per site (Figure 5). The *Ca*. Liberibacter taxa isolated from *T*. *walkeri* (P1–28) and *S*. *umbelliferum* significantly clustered within *Ca*. L. psyllaurous (also known as *Ca*. L. solanacearum) with 100% bootstrap support (Figure 5).

To further determine if the *Ca*. Liberibacter species from *R*. *capsici* (P1–16) is a distinct *Ca*. Liberibacter species compared to known *Ca*. Liberibacter reference genomes we conducted both DDH and ANI analyses. Pairwise DDH estimates for all reference Liberibacter species genomes and *Ca*. L. capsica ranged from 18.9 to 31.1% (Supplementary Table 9). These values are well below the 79% cutoff commonly applied to members of the same subspecies and the 70% cutoff applied to members of the same species. Pairwise ANI estimates for all reference *Ca*. Liberibacter species genomes and *Ca*. L. capsica ranged from 69.72 to 84.49% (Supplementary Table 10). These values are well below the 95% cutoff commonly applied to members of the same species for ANI analyses (Goris et al., 2007). Collectively, these results provide further support that *Ca*. L. capsica (P1–16) is a distinct *Ca*. Liberibacter species.

### *Candidatus* Liberibacter Taxa Is Detected in Additional Psyllid Specimens of *Trichocherms Walkeri*

The purpose of the *Ca*. Liberibacter screening was to (1) determine if the *Ca*. Liberibacter infection is prevalent in the psyllid species that were identified with *Ca*. Liberibacter OTUs from 16S rRNA gene amplicon sequencing, and (2) if the *Ca*. Liberibacter infection occurs in psyllid species that share the same plant host genus as *T*. *walkeri* (*Rhamnus*); samples screened were based on sample availability, see “Materials and Methods” and Supplementary Table 4. Therefore, all DNA extracts from these latter psyllid specimens were screened with the primer set LG774F/LG1463R, which universally amplifies ∼0.7 kb of *Ca*. Liberibacter 16S rRNA fragments (see “Materials and Methods”). Only *T*. *walkeri* samples (*N* = 9, Supplementary Table 4) produced a positive PCR band using this primer set, and therefore were cloned and Sanger sequenced one PCR clone from each sample for BLAST analysis.

Based on NCBI BLAST results, the best BLAST hit (*E*-value = 0) from the nine *T*. *walkeri* samples was the taxon *Ca*. L. psyllaurous with >97% sequence identity (Supplementary Table 11). Although all the *T*. *walkeri* samples matched *Ca*. L. psyllaurous with high sequence identity, there was small nucleotide differences between sample sequences, which may be attributed to the multiple 16S rRNA operon copies within a single *Ca*. Liberibacter genome and/or strain differences within or among psyllid individuals (Supplementary Table 11). These screening results, in terms of identity (*Ca*. L. psyllaurous) and sequence variation, are consistent with 16S rRNA amplicon sequencing and metagenomic sequencing from the *T*. *walkeri* (P1–28) sample, and in turn, this may indicate that *Ca*. Liberibacter psyllaurous is highly prevalent in some *T*. *walkeri* populations.

## Discussion

Our study provides the most taxonomically and geographically diverse dataset of psyllid endosymbiont associations to date, covering 44 divergent taxa across five families. Our results suggest that psyllids house minimal bacterial communities, in agreement with more regionally focused studies of psyllid microbiomes and the microbiomes of other hemipterans (Thao et al., 2000a; Jing et al., 2014; Overholt et al., 2015; Morrow et al., 2017, 2020; Nakabachi et al., 2020a). Similar to the findings of Morrow et al. (2017), we found that bacteria in the class *Enterobacteriaceae* are particularly prevalent in psyllid microbiomes (Figure 1 and Supplementary Table 5). We also found that most sequences recovered during microbiome profiling closely match NCBI GenBank sequences of other insect endosymbionts (Supplementary Table 5). This result indicates that the majority of microbial sequences detected in our study are those of actual psyllid endosymbionts rather than transient associates or contaminants from the environment. Using the genomic data generated through microbiome profiling, we also uncovered putative long-term endosymbiont associations in the psyllid families Triozidae and Psyllidae based on signatures of long-term endosymbiont evolution. Moreover, we identified three psyllid species that are potential hosts for *Ca*. Liberibacter taxa, as well as a new “*Candidatus*” Liberibacter species from the psyllid *R*. *capsici*, for which we propose the name “*Candidatus*” Liberibacter capsica after the psyllid host it was isolated from.

Long-term insect endosymbionts are generally characterized by possessing higher evolutionary rates, lower GC content, and co-cladogenesis patterns with their insect host (Heddi et al., 1998; Nováková et al., 2009). Sap-feeding insects that belong to Hemiptera within the suborder Auchenorrhyncha epitomize very ancient and complex co-occurring endosymbiont associations (Koga et al., 2013). In these latter cases, at least two endosymbionts are obligate for auchenorrhynchan host survival, and losses of some of these long-term endosymbionts occur, and are replaced with a more recent endosymbiont association (Koga et al., 2013). Based on genome content of long-term psyllid symbiont genomes at least three endosymbiont taxa are expected to be long-term endosymbiont associates of psyllids, but these symbiont taxa are not closely related to one another and belong to divergent psyllid lineages (Sloan and Moran, 2012; Nakabachi et al., 2020a,b). Here, we identify two clades (defined here as clade 1 and 2) in *Enterobacteriaceae* that appear to be congruent with two families of psyllids, Triozidae and Psyllidae, respectively (Figure 3). The GC content of clade 1 and 2 is an average of 42 and 45% GC, respectively, which is in between the average range of known obligate and long-term insect endosymbiont sequences (∼41–49%) examined in this study, which primarily belong to Gammaproteobacteria (Figure 2).

Bacterial sequences within clade 1 and 2 had the same best BLAST hit ∼90% sequence identity (within the top three best BLAST hits for two psyllid species) to an already characterized symbiont in Triozidae (“Secondary endosymbiont of *Trioza magnoliae*”) and Psyllidae (“Y-symbiont of *Anomoneura mori*”), respectively (Supplementary Table 5 and Figure 3). Interestingly, the “Y-symbiont of *Anomoneura mori*” was previously characterized to be localized inside the bacteriome (i.e., specialized insect organ to house endosymbionts) of *A*. *mori* within the syncytium of the bacteriome with *Carsonella* being localized within the bacteriocytes in the bacteriome (Fukatsu and Nikoh, 1998). Other long-term obligate endosymbionts of insects including *Profftella* have very similar localization patterns in bacteriomes (Nakabachi et al., 2013). Surprisingly, we did not find a related symbiont to the “Secondary endosymbiont of *Trioza magnoliae*” in *T*. *magnoliae* from our sample as it possessed very high levels of *Wolbachia* contigs, similar to what Morrow et al. (2017) observed for this psyllid species in addition to *Diaphorina citri* in their study. In turn, we conducted a post hoc analysis of *T*. *magnoliae* and found that this *Enterobacteriaceae* endosymbiont was present in our dataset before we filtered out OTUs with low contig coverage, and these sequences had a best BLAST hit with 99% sequence identity with 100% coverage to the “Secondary endosymbiont of *Trioza magnoliae*.” During our post hoc analysis of filtered microbiome reads we also recovered one other psyllid species within Triozidae (*Trioza erytreae*) that had a best BLAST hit to the “Secondary endosymbiont of *Trioza magnoliae*” with ∼90% sequence identity. Analogously, when we searched for filtered microbiome sequences within Psyllidae, sequences from *Cacopsylla saliceti* gave a best BLAST hit to the “Y-symbiont of *Anomoneura mori*” with ∼97% sequence identity. In turn, our dataset is very conservative in regard to the presence of symbiont taxa in specific psyllid species. Therefore, if symbionts within clades 1 and 2 have a congruent relationship with psyllid lineages within Triozidae and Psyllidae, respectively, the remaining psyllid species in these lineages where we did not detect these latter long-term symbionts could have either lost their long-term symbiont or alternatively our sampling effort was not sensitive enough to detect the presence of these symbionts. Understanding putative long-term endosymbiont associations will require more screening of Triozidae and Psyllidae with targeted primers, as well as fluorescence in situ hybridization studies to identify symbiont locations in bacteriomes.

Previous phylogenetic studies on psyllid symbiont taxa have established that numerous facultative symbionts have been horizontally transmitted recently in psyllid lineages (Thao et al., 2000a; Hall et al., 2016; Morrow et al., 2017). Here we identified similar patterns of rampant horizontal transmission in *Enterobacteriaceae* and *Wolbachia* among all psyllid families analyzed here (Figure 3 and Supplementary Figure 2). This was especially the case for OTUs that occurred in more than one psyllid species including the widespread *Wolbachia* lineage (OTU1) (Supplementary Figure 2). For example, *Wolbachia* OTU1 is present in 19 divergent psyllid species spanning across five psyllid families (Supplementary Figure 1). These latter results indicate that a recent divergence of the *Wolbachia* OTU1 lineage has occurred (as an OTU consists of 16S rRNA sequences with 97% sequence identity or higher), and this *Wolbachia* lineage was acquired multiple times in divergent psyllid taxa (Supplementary Figure 2). Very similar findings of recent endosymbiont divergence and horizontal transmission of *Ca*. Hamiltonella defensa, *Ca*. Regiella insecticola, and *Ca*. Serratia symbiotica have also been observed among highly divergent insect species (Russell et al., 2003; Moran et al., 2005).

The most well-known facultative symbionts of psyllids that are of economic concern are those in the genus *Ca*. Liberibacter because many are associated with disease in psyllid plant hosts (Wang et al., 2017; Mauck et al., 2019). In plants, *Ca*. Liberibacter taxa infect phloem vascular tissue and several *Ca*. Liberibacter taxa have emerged as serious threats to food production in the last 100 years (Wang et al., 2017). Interestingly, some *Ca*. Liberibacter taxa, such as *Ca*. L. psyllaurous may actually benefit the psyllid host by suppressing plant defenses that are directed toward the psyllid (Casteel et al., 2012). *Ca*. Liberibacter primarily is an unculturable bacterium which belongs to the Alphaproteobacteria class of bacteria in the phylum Proteobacteria (Bové, 2006). Currently, eight species of *Ca*. Liberibacter have been identified around the world, mostly because of emergence as disease causing agents in crops, and these include *Ca*. L. psyllaurous, *Ca*. L. asiaticus, *Ca*. L. africanus, *Ca*. L. americanus, *Ca*. L. europaeus, *Ca*. L. brunswickensis, *Ca*. L. ctenarytainae, and *L*. *crescens* (Mauck et al., 2019). Based on comprehensive phylogenetic analyses, DDH, ANI and BLAST analyses here, we discovered a new *Ca*. Liberibacter species. The closest relative of this latter new species is *Ca*. L. americanus (Supplementary Tables 5, 9, 10 and Figures 4, 5). This new *Ca*. Liberibacter species, *Ca*. L. capsica, is harbored in *R*. *capsici*, which was collected from Brazil (Supplementary Table 1). *R*. *capsici* has been reported from three localities in Argentina and Brazil so far, including this study, and it is known as a pest of peppers (*Capsicum annuum*) (Burckhardt et al., 2012).

Although it is not known if *R*. *capsici* is a vector for *Ca*. L. capsica, this psyllid species is an emerging pest of concern and has strong potential to invade new areas based on its crop host range (Burckhardt et al., 2012; Syfert et al., 2017). If *Ca*. L. capsica is plant-infecting, pepper and other solanaceous crop plants fed upon by the putative vector may be susceptible to infection. This host’s range is similar to that of *Ca*. L. psyllaurous, which is transmitted primarily by the potato psyllid, *Bactericera cockerelli*, and infects crops (tomato, potato, and pepper) and native plants in the genus *Solanum* (Hansen et al., 2008; Munyaneza et al., 2009; Thinakaran et al., 2015a,b). However, our phylogenetic analysis, DDH, and ANI analyses indicate that *Ca*. L. psyllaurous is not closely related to *Ca*. L. capsica (Supplementary Tables 5, 9, 10 and Figures 4, 5). Instead, the closest relative to *Ca*. L. capsica, based on our phylogenetic, DDH, ANI, and BLAST analyses is *Ca*. L. americanus (Supplementary Tables 5, 9, 10 and Figures 4, 5), which is also found in Brazil. *Ca*. L. americanus is only known to naturally infect plants in the genus *Citrus* (family Rutaceae) and is transmitted by *Diaphorina citri* (Teixeira et al., 2005). However, *Ca*. L. americanus can be transmitted from citrus to a solanaceous host, *Nicotiana tabacum* cv. Xanthi, in the laboratory using the parasitic plant *Cuscuta* (Francischini et al., 2007).

We also detected additional *Ca*. Liberibacter taxa in microbiomes of the psyllid species *T*. *walkeri* (family Triozidae) and *M*. *gladiata* (family Carsidaridae). Subsequent phylogenetic analyses of the *Ca*. Liberibacter species from *T*. *walkeri* suggest that this *Ca*. Liberibacter taxon is nested within *Ca*. L. psyllaurous (Supplementary Tables 5, 11 and Figures 4, 5). The psyllid species *T*. *walkeri* specializes on the woody shrub, *Rhamnus cathartica* (family Rhamnaceae) (McLean, 1998) and specimens for the present study, including the nine additional samples that were positive for *Ca*. L. psyllaurous, were collected in the United Kingdom (Supplementary Tables 1, 4). The species *Ca*. L. psyllaurous is known to establish facultative infections in psyllids within the *Bactericera* and *Trioza* genera and has recently been detected in two *Craspedolepta* species (family Aphalaridae) collected in the United Kingdom (Sumner-Kalkun et al., 2020). *Ca.* L. psyllaurous has not been previously recorded in association with *T*. *walkeri* or other psyllids that feed primarily on woody shrubs or trees. Interestingly, *Ca*. L. psyllaurous has the widest known host plant range of any *Ca.* Liberibacter species to date, however, it is only known to infect herbaceous hosts in the Solanaceae, Apiaceae, and Urticaceae (Nelson et al., 2011; Hajri et al., 2017; Haapalainen et al., 2018). If *T*. *walkeri* is a vector for the new variant of *Ca*. L. psyllaurous detected in this study, it may be able to transmit this pathogen to *Rhamnus* hosts, where it could subsequently establish local or systemic infections.

*Candidatus* Liberibacter sequences were also detected from the psyllid microbiome of *M*. *gladiata*, which was collected from Taiwan (Supplementary Table 1). Unfortunately, the DNA from our specimen was of insufficient quantity to produce a high-quality draft metagenomic assembly of this *Ca.* Liberibacter taxon for phylogenetic analyses using shared orthologs. Nevertheless, the 16S rRNA sequence that we obtained shares 98% sequence similarity to *Ca*. L. asiaticus based on BLAST and 16S rRNA analyses indicates that this taxon is most likely *Ca*. L. asiaticus or a lineage that diverged only very recently from *Ca*. L. asiaticus. *Candidatus* L. asiaticus is one of the main causal agents of the devastating citrus disease, Huanglongbing (HLB) (Bové, 2006). Only two other psyllids, *D*. *citri* and *Cacopsylla citrisuga*, are known to harbor *Ca*. L. asiaticus and they both feed primarily on citrus trees (Sapindales: Rutaceae) (Cen et al., 2012). In contrast, *M*. *gladiata* feeds primarily on *Ficus* species, including cultivated figs (*Ficus carica*) (Rung, 2016). *Ficus* may seem like an unlikely host for *Ca*. L. asiaticus, but it has been recorded as an alternative host for the vector, *Diaphorina citri*, in Florida (where HLB is now endemic), with evidence that it may also support *D*. *citri* reproduction (Thomas and De León, 2011). Use of *Ficus* by *D*. *citri* has not been explored extensively outside of this one study, but several other recent reports document use of various alternative hosts by *D*. *citri* adults, likely as a source of water (Zhang et al., 2019; George et al., 2020). These ecological studies of *D*. *citri* host use suggest there are pathways for rare *Ca*. L. asiaticus horizontal transmission to new psyllid hosts under the right circumstances. In this case, since *M*. *gladiata* is a pest of cultivated *Ficus* species, our detection of a putative *Ca*. L. asiaticus taxon in specimens of this psyllid raises the possibility that a variant of this pathogen could emerge as a new disease-causing agent in figs.

In summary, we documented evidence of new putative long-term psyllid symbiont associations in the Triozidae and Psyllidae, as well as more recent horizontal acquisitions of symbionts belonging to *Enterobacteriaceae* and *Wolbachia*. Additionally, we identified a novel species of *Ca*. Liberibacter associated with an emerging psyllid pest, detected a new association between *Ca*. L. psyllaurous and a psyllid that feeds on woody plants, and detected a potential new association between a *Ca*. L. asiaticus related taxon and a psyllid pest of figs. When examined in the context of psyllid host ecology, host range, and distribution, these findings suggest that expanded microbiome profiling of psyllids has great potential for revealing potential risk of new pathogen development and regulatory concerns. Future studies are needed to further understand the importance of these new psyllid-symbiont relationships of ecological and agricultural importance.

## Data Availability Statement

Raw 16S rRNA amplicon and metagenomic sequencing data are available at NCBI under SRA BioProject IDs PRJNA612536/PRJNA744186. Partial 16S rRNA sequences from the additional *Trichochermes walkeri* samples are deposited to NCBI Genbank with accession numbers OK047422-OK047430.

## Author Contributions

AH helped to design the study, conducted the data and bioinformatic analyses, and helped write the manuscript. YK conducted the data and phylogenetic analyses and helped write the manuscript. VM conducted the bioinformatic analyses of 16S rRNA amplicons, metagenomic assemblies, annotation, and phylogenetic analyses, and helped write the manuscript. KM helped to design the study and helped write the manuscript. DP collected, extracted, and identified all psyllid samples for this study, conducted the psyllid phylogenetic analyses, and helped write the manuscript. PS constructed 16S rRNA libraries, collected the herbarium specimen included in metagenomic sequencing, and performed DNA extractions on this specimen. All authors contributed to the article and approved the submitted version.

## Conflict of Interest

The authors declare that the research was conducted in the absence of any commercial or financial relationships that could be construed as a potential conflict of interest.

## Publisher’s Note

All claims expressed in this article are solely those of the authors and do not necessarily represent those of their affiliated organizations, or those of the publisher, the editors and the reviewers. Any product that may be evaluated in this article, or claim that may be made by its manufacturer, is not guaranteed or endorsed by the publisher.

## Footnotes

1
http://www.earthmicrobiome.org/protocols-and-standards/16s/

2
http://ggdc.dsmz.de/ggdc.php#

3
http://enve-omics.ce.gatech.edu/ani/index

## References

[B1] Andrews S. (2010). *FastQC: A Quality Control Tool for High Throughput Sequence Data.* Available online at: http://www.bioinformatics.babraham.ac.uk/projects/fastqc/ (accessed July 10, 2019).

[B2] Baumann P. (2005). Biology bacteriocyte-associated endosymbionts of plant sap-sucking insects. *Annu. Rev. Microbiol.* 59 155–189. 10.1146/annurev.micro.59.030804.121041 16153167

[B3] Bolger A. M. Lohse M. Usadel B. (2014). Trimmomatic: a flexible trimmer for Illumina sequence data. *Bioinformatics* 30 2114–2120. 10.1093/bioinformatics/btu170 24695404 PMC4103590

[B4] Bové J. M. (2006). Huanglongbing: a destructive, newly-emerging, century-old disease of citrus. *J. Plant Pathol.* 88 7–37. Available online at: http://www.sipav.org/main/jpp/index.php/jpp/article/view/828/615

[B5] Buchner P. (1965). *Endosymbiosis of Animals With Plant Microorganisms.* New York: Interscience Publishers.

[B6] Burckhardt D. Ouvrard D. Percy D. M. (2021). An updated classification of the jumping plant-lice (Hemiptera: Psylloidea) integrating molecular and morphological evidence. *EJT* 736 137–182. 10.5852/ejt.2021.736.1257

[B7] Burckhardt D. Queiroz D. L. Rezende M. Q. Castro de Queiroz E. Bouvet J. P. (2012). The capsicum psyllid, Russelliana capsici (Hemiptera, Psylloidea), a pest on Capsicum annuum (Solanaceae) in Argentina and Brazil. *Mitt. Schweiz. Entomol. Ges.* 85, 71–78.

[B8] Bushnell B. (2014). *BBMap: A Fast, Accurate, Splice-Aware Aligner.* Berkeley, CA: Lawrence Berkeley National Lab.(LBNL).

[B9] Capella-Gutiérrez S. Silla-Martínez J. M. Gabaldón T. (2009). trimAl: a tool for automated alignment trimming in large-scale phylogenetic analyses. *Bioinformatics* 25 1972–1973. 10.1093/bioinformatics/btp348 19505945 PMC2712344

[B10] Caporaso J. G. Lauber C. L. Walters W. A. Berg-Lyons D. Huntley J. Fierer N. (2012). Ultra-high-throughput microbial community analysis on the Illumina HiSeq and MiSeq platforms. *ISME J.* 6 1621–1624. 10.1038/ismej.2012.8 22402401 PMC3400413

[B11] Casteel C. L. Hansen A. K. (2014). Evaluating insect-microbiomes at the plant-insect interface. *J. Chem. Ecol.* 40 836–847. 25052911 10.1007/s10886-014-0475-4

[B12] Casteel C. L. Hansen A. K. Walling L. L. Paine T. D. (2012). Manipulation of plant defense responses by the tomato psyllid (*Bactericerca cockerelli*) and its associated endosymbiont *Candidatus* Liberibacter psyllaurous. *PLoS One* 7:e35191. 10.1371/journal.pone.0035191 22539959 PMC3335145

[B13] Cen Y. Zhang L. Xia Y. Guo J. Deng X. Zhou W. (2012). Detection of ‘*Candidatus* Liberibacter asiaticus’ in *Cacopsylla (Psylla) citrisuga* (Hemiptera: Psyllidae). *Florida Entomol.* 95 304–311.

[B14] Douglas A. E. (2009). The microbial dimension in insect nutritional ecology. *Functional Ecol.* 23 38–47. 10.1111/j.1365-2435.2008.01442.x

[B15] Emms D. M. Kelly S. (2015). OrthoFinder: solving fundamental biases in whole genome comparisons dramatically improves orthogroup inference accuracy. *Genome Biol.* 16:157. 10.1186/s13059-015-0721-2 26243257 PMC4531804

[B16] Feldhaar H. (2011). Bacterial symbionts as mediators of ecologically important traits of insect hosts. *Ecol. Entomol.* 36 533–543. 10.1111/j.1365-2311.2011.01318.x

[B17] Francischini F. J. B. Oliveira K. D. S. Astúa-Monge G. Novelli A. Lorenzino R. Matiolli C. (2007). First report on the transmission of “*Candidatus* Liberibacter americanus” from citrus to *Nicotiana tabacum* cv. Xanthi. *Plant Dis.* 91 631–631. 10.1094/PDIS-91-5-0631B 30780712

[B18] Fukatsu T. Nikoh N. (1998). Two intracellular symbiotic bacteria from the mulberry psyllid *Anomoneura mori* (insecta, homoptera). 64 3599–3606. 10.1128/AEM.64.10.3599-3606.1998 9758773 PMC106470

[B19] George J. Kanissery R. Ammar E.-D. Cabral I. Markle L. T. Patt J. M. (2020). Feeding behavior of asian citrus psyllid [*Diaphorina citri* (Hemiptera: Liviidae)] nymphs and adults on common weeds occurring in cultivated citrus described using electrical penetration graph recordings. *Insects* 11:48. 10.3390/insects11010048 32284515 PMC7023154

[B20] Goris J. Konstantinidis K. T. Klappenbach J. A. Coenye T. Vandamme P. Tiedje J. M. (2007). DNA-DNA hybridization values and their relationship to whole-genome sequence similarities. *Int. J. Syst. Evol. Microbiol.* 57 81–91. 10.1099/ijs.0.64483-0 17220447

[B21] Haapalainen M. Wang J. Latvala S. Lehtonen M. T. Pirhonen M. Nissinen A. I. (2018). Genetic variation of “*Candidatus* Liberibacter solanacearum” haplotype C and identification of a novel haplotype from trioza urticae and stinging nettle. *Phytopathology* 108 925–934. 10.1094/PHYTO-12-17-0410-R 29600888

[B22] Hajri A. Loiseau M. Cousseau-Suhard P. Renaudin I. Gentit P. (2017). Genetic characterization of “*Candidatus* Liberibacter solanacearum” haplotypes associated with apiaceous crops in france. *Plant Dis.* 101 1383–1390. 10.1094/PDIS-11-16-1686-RE 30678593

[B23] Hall A. A. G. Morrow J. L. Fromont C. Steinbauer M. J. Taylor G. S. Johnson S. N. (2016). Codivergence of the primary bacterial endosymbiont of psyllids versus host switches and replacement of their secondary bacterial endosymbionts. *Environ. Microbiol.* 18 2591–2603. 10.1111/1462-2920.13351 27114069

[B24] Hansen A. Trumble J. Stouthamer R. Paine T. (2008). A new huanglongbing species,“*Candidatus* Liberibacter psyllaurous,” found to infect tomato and potato, is vectored by the psyllid *Bactericera cockerelli* (Sulc). *Appl. Environ. Microbiol.* 74 5862–5865. 18676707 10.1128/AEM.01268-08 PMC2547047

[B25] Hansen A. K. Moran N. A. (2014). The impact of microbial symbionts on host plant utilization by herbivorous insects. *Mol. Ecol.* 23 1473–1496. 10.1111/mec.12421 23952067

[B26] Heddi A. Charles H. Khatchadourian C. Bonnot G. Nardon P. (1998). Molecular characterization of the principal symbiotic bacteria of the weevil *Sitophilus oryzae*: a peculiar G + C content of an endocytobiotic DNA. *J. Mol. Evol.* 47 52–61. 10.1007/pl00006362 9664696

[B27] Jing X. Wong A. C.-N. Chaston J. M. Colvin J. McKenzie C. L. Douglas A. E. (2014). The bacterial communities in plant phloem-sap-feeding insects. *Mol. Ecol.* 23 1433–1444. 10.1111/mec.12637 24350573

[B28] Katoh K. Standley D.M. (2013). MAFFT multiple sequence alignment software version 7: improvements in performance and usability. *Mol. Biol. Evol.* 30, 772–780. 10.1093/molbev/mst010 23329690 PMC3603318

[B29] Koga R. Bennett G. M. Cryan J. R. Moran N. A. (2013). Evolutionary replacement of obligate symbionts in an ancient and diverse insect lineage. *Environ. Microbiol.* 15 2073–2081. 10.1111/1462-2920.12121 23574391

[B30] Kozich J. J. Westcott S. L. Baxter N. T. Highlander S. K. Schloss P. D. (2013). Development of a dual-index sequencing strategy and curation pipeline for analyzing amplicon sequence data on the MiSeq Illumina sequencing platform. *Appl. Environ. Microbiol.* 79 5112–5120. 10.1128/AEM.01043-13 23793624 PMC3753973

[B31] Lambert J. D. Moran N. A. (1998). Deleterious mutations destabilize ribosomal RNA in endosymbiotic bacteria. *Proc. Natl. Acad. Sci. U.S.A.* 95 4458–4462. 10.1073/pnas.95.8.4458 9539759 PMC22511

[B32] Li D. Liu C.-M. Luo R. Sadakane K. Lam T.-W. (2015). MEGAHIT: an ultra-fast single-node solution for large and complex metagenomics assembly via succinct de Bruijn graph. *Bioinformatics* 31 1674–1676. 10.1093/bioinformatics/btv033 25609793

[B33] Maddison W. Maddison D. (2018). *Mesquite: A Modular System for Evolutionary Analysis. Version 3.51.*

[B34] Mauck K. E. Sun P. Meduri V. R. Hansen A. K. (2019). New *Ca.* Liberibacter psyllaurous haplotype resurrected from a 49-year-old specimen of *Solanum umbelliferum*: a native host of the psyllid vector. *Sci. Rep.* 9 1–13. 10.1038/s41598-019-45975-6 31267035 PMC6606623

[B35] McFrederick Q. S. Rehan S. M. (2016). Characterization of pollen and bacterial community composition in brood provisions of a small carpenter bee. *Mol. Ecol.* 25 2302–2311. 10.1111/mec.13608 26945527

[B36] McLean I. F. G. (1998). “The population ecology of *Trichochermes walkeri*,” in *Insect Populations In theory and in practice: 19th Symposium of the Royal Entomological Society 10–11 September 1997 at the University of Newcastle*, eds Dempster J. P. McLean I. F. G. (Dordrecht: Springer Netherlands), 341–366. 10.1007/978-94-011-4914-3_15

[B37] Meier-Kolthoff J. P. Auch A. F. Klenk H.-P. Göker M. (2013). Genome sequence-based species delimitation with confidence intervals and improved distance functions. *BMC Bioinform.* 14:60. 10.1186/1471-2105-14-60 23432962 PMC3665452

[B38] Moran N. A. (2007). Symbiosis as an adaptive process and source of phenotypic complexity. *Proc. Natl. Acad. Sci. U.S.A.* 104 8627–8633. 10.1073/pnas.0611659104 17494762 PMC1876439

[B39] Moran N. A. Bennett G. M. (2014). The tiniest tiny genomes. *Annu. Rev. Microbiol.* 68, 195–215. 10.1146/annurev-micro-091213-112901 24995872

[B40] Moran N. A. Russell J. A. Koga R. Fukatsu T. (2005). Evolutionary relationships of three new species of *Enterobacteriaceae* living as symbionts of aphids and other insects. *Appl. Environ. Microbiol.* 71 3302–3310. 10.1128/AEM.71.6.3302-3310.2005 15933033 PMC1151865

[B41] Morris J. Shiller J. Mann R. Smith G. Yen A. Rodoni B. (2017). Novel “*Candidatus* Liberibacter” species identified in the Australian eggplant psyllid, *Acizzia solanicola*. *Microb. Biotechnol.* 10 833–844. 10.1111/1751-7915.12707 28387006 PMC5481521

[B42] Morrow J. L. Hall A. A. G. Riegler M. (2017). Symbionts in waiting: the dynamics of incipient endosymbiont complementation and replacement in minimal bacterial communities of psyllids. *Microbiome* 5:58. 10.1186/s40168-017-0276-4 28587661 PMC5461708

[B43] Morrow J. L. Om N. Beattie G. A. C. Chambers G. A. Donovan N. J. Liefting L. W. (2020). Characterization of the bacterial communities of psyllids associated with Rutaceae in Bhutan by high throughput sequencing. *BMC Microbiol.* 20:1895. 10.1186/s12866-020-01895-4 32689950 PMC7370496

[B44] Munyaneza J. E. Sengoda V. G. Crosslin J. M. Garzón-Tiznado J. A. Cardenas-Valenzuela O. G. (2009). First report of “*Candidatus* Liberibacter solanacearum” in Tomato Plants in México. *Plant Dis.* 93 1076–1076. 10.1094/PDIS-93-10-1076A 30754366

[B45] Eren A. M. Esen ÖC. Quince C. Vineis J. H. Morrison H. G. Sogin M. L. (2015). Anvi’o: an advanced analysis and visualization platform for ‘omics data. *PeerJ* 3:e1319. 10.7717/peerj.1319 26500826 PMC4614810

[B46] Nachappa P. Levy J. Pierson E. Tamborindeguy C. (2011). Diversity of endosymbionts in the potato psyllid, *Bactericera cockerelli* (Hemiptera: Triozidae), vector of zebra chip disease of potato. *Curr. Microbiol.* 62 1510–1520. 10.1007/s00284-011-9885-5 21327558

[B47] Nadarasah G. Stavrinides J. (2011). Insects as alternative hosts for phytopathogenic bacteria. *FEMS Microbiol. Rev.* 35 555–575. 10.1111/j.1574-6976.2011.00264.x 21251027

[B48] Nakabachi A. Malenovský I. Gjonov I. Hirose Y. (2020a). 16S rRNA sequencing detected *Profftella, Liberibacter, Wolbachia*, and *Diplorickettsia* from relatives of the Asian citrus psyllid. *Microbial. Ecol.* 80 410–422. 10.1007/s00248-020-01491-z 32052099

[B49] Nakabachi A. Piel J. Malenovský I. Hirose Y. (2020b). Comparative genomics underlines multiple roles of *Profftella*, an obligate symbiont of psyllids: providing toxins, vitamins, and carotenoids. *Geno. Biol. Evolu.* 12 1975–1987. 10.1093/gbe/evaa175 32797185 PMC7643613

[B50] Nakabachi A. Ueoka R. Oshima K. Teta R. Mangoni A. Gurgui M. (2013). Defensive bacteriome symbiont with a drastically reduced genome. *Curr. Biol.* 23 1478–1484. 10.1016/j.cub.2013.06.027 23850282

[B51] Nelson W. R. Fisher T. W. Munyaneza J. E. (2011). Haplotypes of “*Candidatus* Liberibacter solanacearum” suggest long-standing separation. *Eur. J. Plant Pathol.* 130 5–12. 10.1007/s10658-010-9737-3

[B52] Nováková E. Hypša V. Moran N. A. (2009). *Arsenophonus*, an emerging clade of intracellular symbionts with a broad host distribution. *BMC Microbiol.* 9:1–14. 10.1186/1471-2180-9-143 19619300 PMC2724383

[B53] Oliver K. M. Degnan P. H. Burke G. R. Moran N. A. (2010). Facultative symbionts in aphids and the horizontal transfer of ecologically important traits. *Annu. Rev. Entomol.* 55 247–266. 10.1146/annurev-ento-112408-085305 19728837

[B54] Overholt W. A. Diaz R. Rosskopf E. Green S. J. Overholt W. A. (2015). Deep Characterization of the Microbiomes of *Calophya* spp. (Hemiptera: Calophyidae) gall-inducing psyllids reveals the absence of plant pathogenic bacteria and three dominant endosymbionts. *PLoS One* 10:e0132248. 10.1371/journal.pone.0132248 26161659 PMC4498736

[B55] Paradis E. Claude J. Strimmer K. (2004). APE: analyses of phylogenetics and evolution in R language. *Bioinformatics* 20 289–290. 14734327 10.1093/bioinformatics/btg412

[B56] Percy D. M. Crampton-Platt A. Sveinsson S. Lemmon A. R. Lemmon E. M. Ouvrard D. (2018). Resolving the psyllid tree of life: phylogenomic analyses of the superfamily Psylloidea (Hemiptera). *Syst. Entomol.* 43 762–776. 10.1111/syen.12302

[B57] R Core Team (2021). *R: A Language and Environment for Statistical Computing.* Vienna: R Foundation for Statistical Computing.

[B58] Raddadi N. Gonella E. Camerota C. Pizzinat A. Tedeschi R. Crotti E. (2011). ‘*Candidatus* Liberibacter europaeus’ sp. nov. that is associated with and transmitted by the psyllid *Cacopsylla pyri* apparently behaves as an endophyte rather than a pathogen. *Environ. Microbiol.* 13 414–426. 21040355 10.1111/j.1462-2920.2010.02347.x

[B59] Rambaut A. (2018). Available online at: http://tree.bio.ed.ac.uk/software/figtree/, viewed (accessed September 9, 2021)

[B60] Rodriguez R. L. M. Konstantinidis K. T. (2016). The enveomics collection: a toolbox for specialized analyses of microbial genomes and metagenomes. *PeerJ* 4:e1900v1. 10.7287/peerj.preprints.1900v1

[B61] Rung A. (2016). A new pest of ficus in California: *Macrohomotoma gladiata* Kuwayama, 1908 (Hemiptera: Psylloidea: Homotomidae), new to North America. *CheckList* 12 1–5. 10.15560/12.3.1882

[B62] Russell J. A. Latorre A. Sabater-Muñoz B. Moya A. Moran N. A. (2003). Side-stepping secondary symbionts: widespread horizontal transfer across and beyond the Aphidoidea. *Mol. Ecol.* 12 1061–1075. 10.1046/j.1365-294x.2003.01780.x 12753224

[B63] Schloss P. D. Westcott S. L. Ryabin T. Hall J. R. Hartmann M. Hollister E. B. (2009). Introducing mothur: open-source, platform-independent, community-supported software for describing and comparing microbial communities. *Appl. Environ. Microbiol.* 75 7537–7541. 10.1128/aem.01541-09 19801464 PMC2786419

[B64] Seemann T. (2014). Prokka: rapid prokaryotic genome annotation. *Bioinformatics* 30 2068–2069. 10.1093/bioinformatics/btu153 24642063

[B65] Sloan D. B. Moran N. A. (2012). Genome reduction and co-evolution between the primary and secondary bacterial symbionts of psyllids. *Mol. Biol. Evolu.* 29 3781–3792. 10.1093/molbev/mss180 22821013 PMC3494270

[B66] Spaulding A. W. von Dohlen C. D. (2001). Psyllid endosymbionts exhibit patterns of co-speciation with hosts and destabilizing substitutions in ribosomal RNA. *Insect Mol. Biol.* 10 57–67. 10.1093/oxfordjournals.molbev.a025878 11240637

[B67] Stackebrandt E. Goebel B. M. (1994). Taxonomic note: a place for DNA-DNA reassociation and 16S rRNA sequence analysis in the present species definition in bacteriology. *Int. J. Syst. Evol. Microbiol.* 44 846–849. 10.1099/00207713-44-4-846

[B68] Stamatakis A. (2014). RAxML version 8: a tool for phylogenetic analysis and post-analysis of large phylogenies. *Bioinformatics* 30 1312–1313. 10.1093/bioinformatics/btu033 24451623 PMC3998144

[B69] Sudakaran S. Kost C. Kaltenpoth M. (2017). Symbiont acquisition and replacement as a source of ecological innovation. *Trends Microbiol.* 25 375–390. 28336178 10.1016/j.tim.2017.02.014

[B70] Sumner-Kalkun J. C. Highet F. Arnsdorf Y. M. Back E. Carnegie M. Madden S. (2020). ‘*Candidatus* Liberibacter solanacearum’distribution and diversity in Scotland and the characterisation of novel haplotypes from *Craspedolepta* spp.(Psyllidae: Aphalaridae). *Sci. Rep.* 10 1–11. 10.1038/s41598-020-73382-9 33024134 PMC7538894

[B71] Syfert M. M. Serbina L. Burckhardt D. Knapp S. Percy D. M. (2017). Emerging new crop pests: ecological modelling and analysis of the South American potato psyllid *Russelliana solanicola* (Hemiptera: Psylloidea) and its wild relatives. *PLoS One* 12:e0167764. 10.1371/journal.pone.0167764 28052088 PMC5214844

[B72] Teixeira D. do C. Saillard C. Eveillard S. Danet J. L. Costa P. I. (2005). “*Candidatus* Liberibacter americanus”, associated with citrus huanglongbing (greening disease) in São Paulo State. *Brazil. Int. J. Syst. Evol. Microbiol.* 55 1857–1862. 10.1099/ijs.0.63677-0 16166678

[B73] Thao M. L. Clark M. A. Baumann L. Brennan E. B. Moran N. A. Baumann P. (2000a). Secondary endosymbionts of psyllids have been acquired multiple times. *Curr. Microbiol.* 41 300–304. 10.1007/s002840010138 10977900

[B74] Thao M. L. Moran N. A. Abbot P. Brennan E. B. Burckhardt D. H. Baumann P. (2000b). Cospeciation of psyllids and their primary prokaryotic endosymbionts. *Appl. Environ. Microbiol.* 66 2898–2905. 10.1128/AEM.66.7.2898-2905.2000 10877784 PMC92089

[B75] Thinakaran J. Pierson E. Kunta M. Munyaneza J. E. Rush C. M. Henne D. C. (2015a). Silverleaf nightshade (*Solanum elaeagnifolium*), a reservoir host for ‘*Candidatus* Liberibacter solanacearum’, the putative causal agent of zebra chip disease of potato. *Plant Dis.* 99 910–915. 10.1094/PDIS-12-14-1254-RE 30690968

[B76] Thinakaran J. Pierson E. Longnecker M. Tamborindeguy C. Munyaneza J. Rush C. (2015b). Settling and ovipositional behavior of *Bactericera cockerelli* (Hemiptera: Triozidae) on solanaceous hosts under field and laboratory conditions. *J. Econ. Entomol.* 108 904–916. 10.1093/jee/tov058 26470210

[B77] Thomas D. B. De León J. H. (2011). Is the old world fig, *Ficus carica* L.(Moraceae), an alternative host for the asian citrus psyllid, *Diaphorina citri* (Kuwayama)(Homoptera: Psyllidae)? *Florida Entomol.* 94 1081–1083. 10.1653/024.094.0455

[B78] Thompson S. Jorgensen N. Bulman S. Smith G. (2017). “A novel Candidatus Liberibacter species associated with Ctenarytaina fuchsiae, the New Zealand native fuchsia psyllid,” in *Proceedings of the Science Protecting Plant Health 2017, 27 Sept. 2017*, Brisbane, QLD.

[B79] Walters W. Hyde E. R. Berg-Lyons D. Ackermann G. Humphrey G. Parada A. (2016). Improved bacterial 16S rRNA gene (V4 and V4-5) and fungal internal transcribed spacer marker gene primers for microbial community surveys. *Msystems* 1 e00009–e00015. 10.1128/mSystems.00009-15 PMC5069754 27822518

[B80] Wang N. Pierson E. A. Setubal J. C. Xu J. Levy J. G. Zhang Y. (2017). The *Candidatus* Liberibacter–host interface: insights into pathogenesis mechanisms and disease control. *Ann. Rev. Phytopathol.* 55 451–482. 28637377 10.1146/annurev-phyto-080516-035513

[B81] Wickham H. (2016). *ggplot2: Elegant Graphics for Data Analysis.* New York: Springer-Verlag.

[B82] Zhang R. He S. Wu W. Huang Y. Zhu C. Xiao F. (2019). Survival and lifespan of *Diaphorina citri* on non-host plants at various temperatures. *Crop Protection* 124:104841.

