# Supplementary figures and images for: Uncovering Symbionts Across the Psyllid Tree of Life and the Discovery of a New Liberibacter Species, “Candidatus” Liberibacter capsica
Source: Front Microbiol. 2021 Sep 29;12:739763. doi: 10.3389/fmicb.2021.739763 (PMC8511784; doi:10.3389/fmicb.2021.739763)

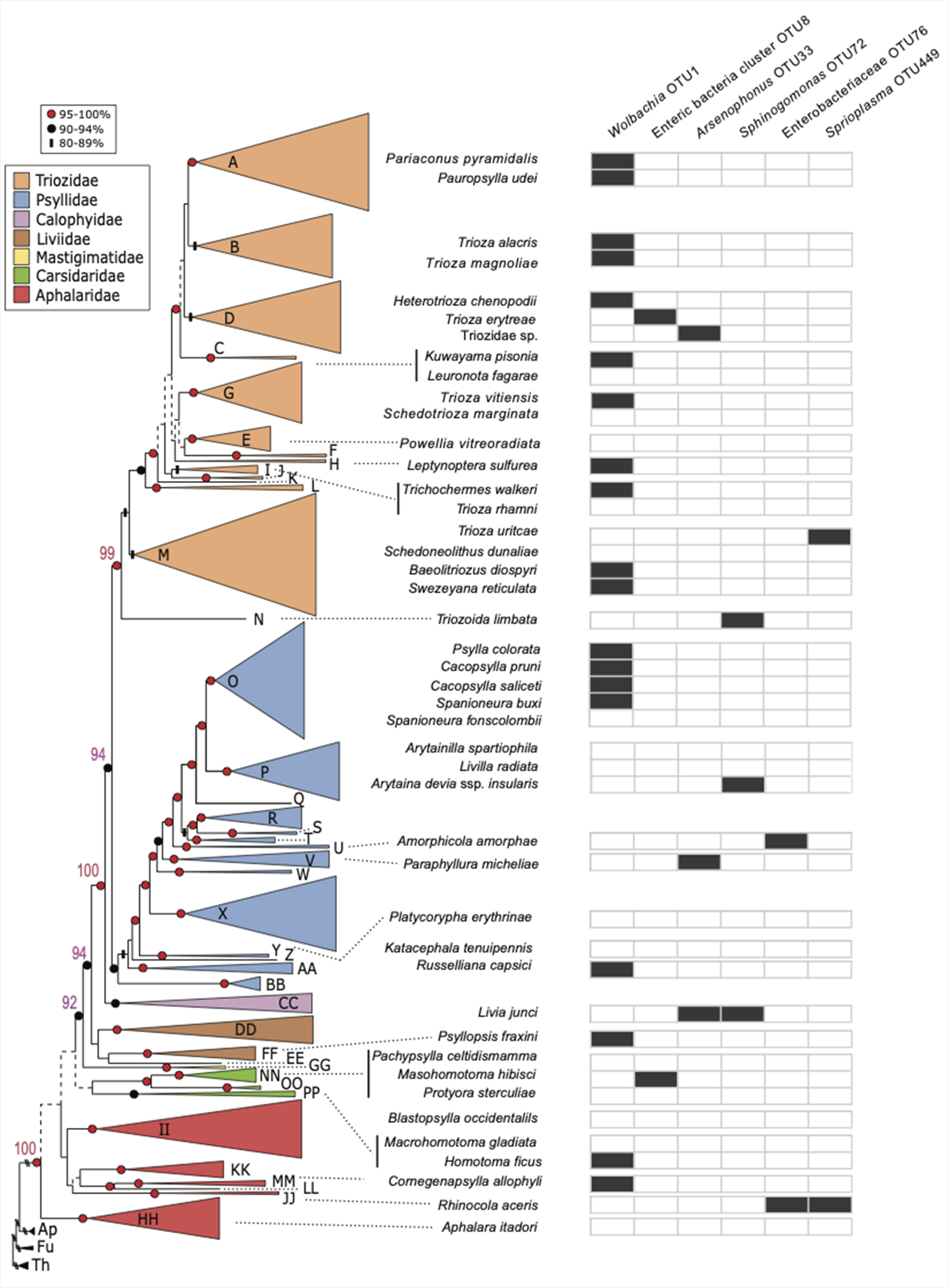

Supplement: Supplementary file 1 [file Image_1.tiff]

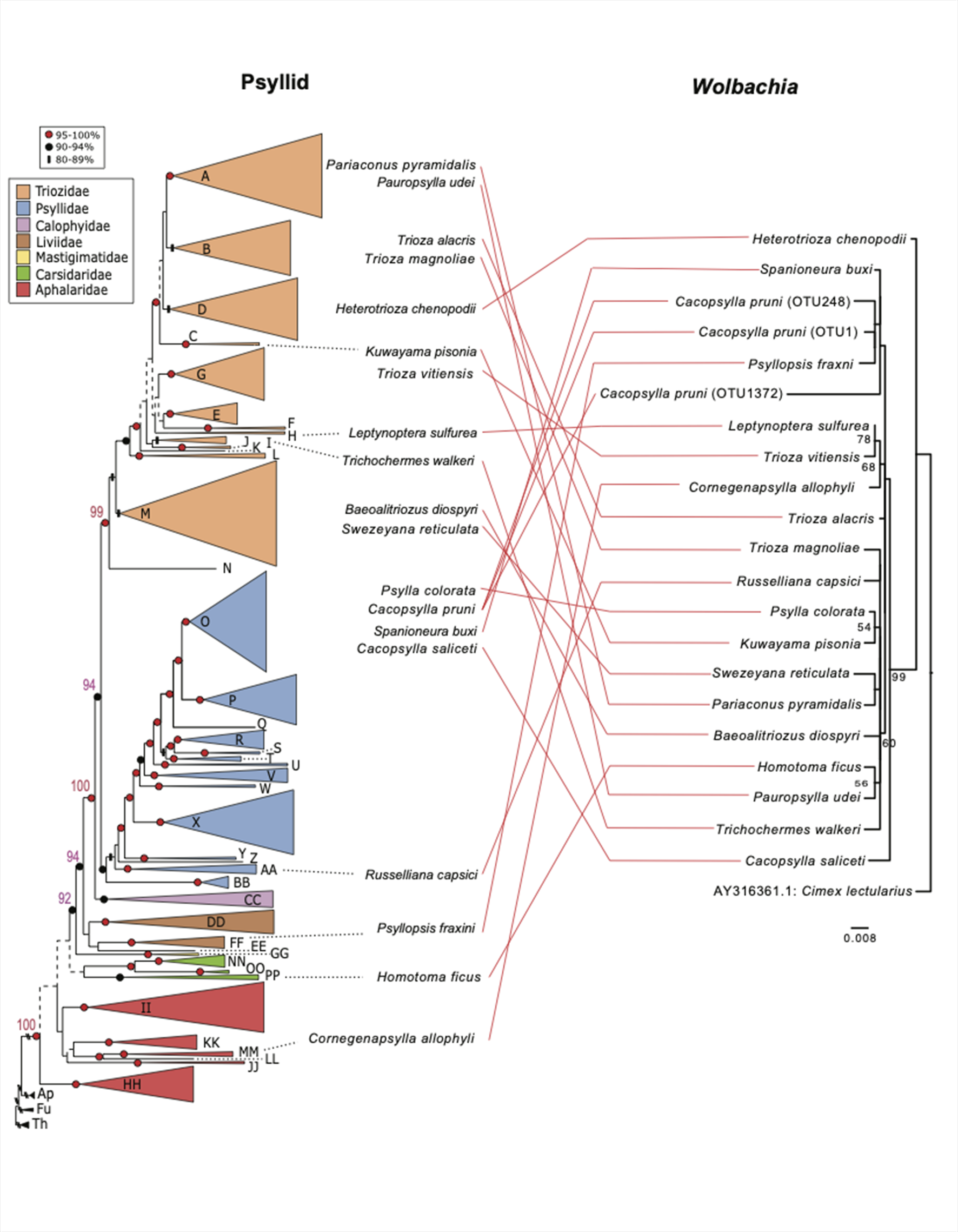

Supplement: Supplementary file 2 [file Image_2.tiff]
