# Supplementary material for: Uncovering Symbionts Across the Psyllid Tree of Life and the Discovery of a New Liberibacter Species, “Candidatus” Liberibacter capsica
Source: Front Microbiol. 2021 Sep 29;12:739763. doi: 10.3389/fmicb.2021.739763 (PMC8511784; doi:10.3389/fmicb.2021.739763)
Supplement: Supplementary file 3 [file Data_Sheet_1.docx]

**Supplementary Figure 1.** Maximum likelihood tree of Psylloidea (left) redrawn from Percy et al. (2018) with updated classification according to Burckhardt et al. (2021), and the presence of OTUs that occur in one or more psyllid species, indicated as black rectangles (right). Capital letters in the psyllid phylogeny represent generic groups presented in Percy et al. (2018) and are detailed in Supplementary Table 1. Node support is indicated by shape and color of node symbols, and nodes with < 50% bootstrap support are indicated by dotted lines. The 44 psyllid species with sequenced 16S rRNA microbiomes in this study are presented in the Psylloidea tree following current taxonomic and generic group classifications based on Percy et al. (2018) and Burckhardt et al. (2021).

**Supplementary Figure 2.** Comparison of phylogenies from both psyllid hosts and their endosymbiont *Wolbachia*. Maximum likelihood tree of Psylloidea (left) redrawn from Percy et al. (2018) with updated classification according to Burckhardt et al. (2021). For the psyllid phylogeny node support is indicated by shape and color of node symbols, and nodes with < 50% bootstrap support are indicated by dotted lines. Capital letters in the psyllid phylogeny represent generic groups presented in Percy et al. (2018) and are detailed in Supplementary Table 1. Psyllid species names from samples in this study that harbor *Wolbachia* with 16S rRNA sequences that have a GC content in the range of ~41-49% are indicated on both the psyllid and *Wolbachia* phylogenies. For the *Wolbachia* 16S rRNA phylogeny (429-nt) using RAxML with 100 bootstraps only branch support at 50% or above is shown. The scale bar indicates nucleotide changes per site. The tree was rooted with the outgroup *Wolbachia* of *Cimex lectularius* (AY316361.1).
